# Diagnostic performance and safety of the edrophonium test in myasthenia gravis: a retrospective case-control study

**DOI:** 10.1186/s42466-026-00468-6

**Published:** 2026-02-09

**Authors:** Omar Keritam, Julia Bettina Kräutler, Diana Gharib, Elnayer Ahmed Jabir, Rosa Weng, Fiona Jäger, Martin Krenn, Anna Paul, Walter Rinner, Hakan Cetin, Fritz Zimprich, Gudrun Zulehner, Jakob Rath

**Affiliations:** 1 Department of Neurology, Medical University of Vienna, Waehringer Guertel 18-20, Vienna, 1090 Austria; 2 Comprehensive Center for Clinical Neurosciences and Mental Health, Medical University of Vienna, Vienna, Austria; 3 Department of Neurosurgery, University Hospital Augsburg, Augsburg, Germany; 4 Neurological Rehabilitation Center (NRZ) Rosenhügel, Vienna, Austria; 5 Department of Neurology, Clinic Ottakring, Vienna, Austria; 6 Department of Neurology, Clinic Floridsdorf, Vienna, Austria

**Keywords:** Tensilon test, Edrophonium test, Diagnostic testing, Myasthenia gravis, Safety, Case-control study

## Abstract

**Background:**

Myasthenia gravis (MG) is an autoimmune disorder of the neuromuscular junction. The diagnosis typically relies on clinical features, serologic testing, and neurophysiological assessment but provocation tests such as the edrophonium test can provide rapid supportive information; however, data on its diagnostic performance are limited. Thus, we aimed to evaluate the diagnostic performance and safety of the edrophonium test in MG.

**Methods:**

We conducted a retrospective case–control study of patients who underwent an edrophonium test at the Department of Neurology of the Medical University of Vienna between January 1991 and January 2024. We calculated sensitivity, specificity, and likelihood ratios and performed a multivariable logistic regression analysis to identify variables associated with a positive edrophonium test. Additionally, we assessed the safety of the edrophonium test.

**Results:**

We included 182 patients with MG (41.2% female; mean age 55.8 years) and 324 controls (55.2% female; mean age 53.6 years). The edrophonium test had a sensitivity of 83.5% and a specificity of 87.7% in diagnosing MG. Patients with a decrement after repetitive nerve stimulation had higher odds of a positive response to the edrophonium test (OR 3.79, 95% CI 1.48–10.33, *p* = 0.0067), while odds were lower in patients with MuSK-MG compared to patients with AChR-MG (OR 0.08, 95% CI 0.01–0.82, *p* = 0.0254). Adverse events were reported in 58 patients (11.5%), in most of whom (53 patients, 91.4%) they were mild.

**Conclusions:**

We provide data on the diagnostic performance and safety of the edrophonium test, supporting its use as an adjunctive diagnostic test for the diagnosis of MG.

**Supplementary Information:**

The online version contains supplementary material available at 10.1186/s42466-026-00468-6.

## Background

Myasthenia gravis (MG) is an autoimmune disorder of the neuromuscular junction caused by autoantibodies against the postsynaptic acetylcholine receptor (AChR) in most cases [1]. Antibodies against the muscle-specific kinase (MuSK) and the low-density lipoprotein-related protein 4 (LRP4) can be detected in a minority of patients. A small proportion of patients remain seronegative (SNMG) on antibody testing [1]. Beyond MG, the autoimmune-mediated Lambert–Eaton myasthenic syndrome can present with similar clinical features, while AChR antibodies are also implicated in the spectrum of fetal acetylcholine receptor antibody–related disorders [2–5]. MG is typically characterised by muscle fatigability and fluctuating muscle weakness with different phenotypes ranging from pure ocular to severe generalised forms [1, 6, 7]. Antibody status, age at onset, and thymic pathology may influence treatment decisions in patients with MG [2, 6, 8, 9]. Together with antibody testing, repetitive nerve stimulation (RNS) or single-fibre electromyography (SFEMG) are recommended as the standard neurophysiological tests for the diagnosis of MG [10–12]. However, RNS and SFEMG are not available in all centres, especially in primary and secondary care facilities. Additionally, they are associated with various limitations, such as a lower sensitivity of RNS in ocular MG or lower specificity of SFEMG for the diagnosis of MG [13–17]. In such cases, bedside tests such as the ice-pack test or pharmacological provocation tests like the edrophonium test may serve as valuable complementary diagnostic tools. Moreover, the edrophonium test can provide immediate information for severely affected patients in whom urgent treatment decisions must be made before antibody-testing results become available. Edrophonium is a short-acting, reversible acetylcholinesterase inhibitor that can rapidly improve myasthenic symptoms. Because rare side-effects such as bradycardia and respiratory failure may occur, patients must be closely monitored during and after the test, and atropine should be kept readily available as an antidote. While the edrophonium test has been used in clinical practice since the 1950s, few studies have evaluated its diagnostic performance reporting sensitivities and specificities in the range of 88–100% and 90–100%, respectively [18–22]. Therefore, we retrospectively evaluated the diagnostic performance and safety profile of the edrophonium test using data collected over 33 years at a tertiary care centre.

## Methods

### Study design and cohort

We conducted a retrospective case–control study of patients who underwent an edrophonium test at the Department of Neurology of the Medical University of Vienna between January 1991 and January 2024. Demographic, clinical and diagnostic data were extracted from the hospital’s electronic database. Diagnosis of MG was based on typical clinical symptoms together with a positive antibody test (elevated AChR antibody titre in a radioimmunoassay [RIA; cut-off ≥ 0.25 nmol/l; applied to all cases], or positivity for antibodies against AChR, MuSK, or LRP4 in cell-based assay [CBA] or in enzyme-linked immunosorbent assay [ELISA]). Additionally, seronegative cases with symptoms of MG were included if a decrement after RNS was found or a response to immunosuppressive treatment at any timepoint after diagnosis was documented. Patients with other rare myasthenic syndromes such as Lambert-Eaton myasthenic syndrome or congenital myasthenic syndromes were excluded. The control group comprised patients without AChR antibodies in whom MG was excluded or a more likely alternative diagnosis was suspected based on the patient history (see supplementary Table 1 for the list of alternative diagnoses). MG subtypes were classified as early-onset AChR-MG (< 50 years), late-onset AChR-MG (≥ 50 years), thymoma-associated AChR-MG, MuSK-MG, LRP4-MG, and SNMG [7]. MG severity at the time of the edrophonium test was classified according to the Myasthenia Gravis Foundation of America (MGFA classes I-V). This study was approved by the ethics committee of the Medical University of Vienna (EK No. 1218/2024). 

### Edrophonium test

The edrophonium test followed a two-step protocol [23], with an initial intravenous administration of 2 mg edrophonium chloride. If no adverse events were observed for approximately 60 s, additional 8 mg were subsequently administered, and clinical response was evaluated by neurologists. Positive clinical responses were subjectively graded by the examining neurologists. For the purposes of this study, positive responses were retrospectively dichotomised as minimal versus moderate/strong based on the descriptions in the clinical reports. Adverse events (AEs) were retrospectively graded using the Common Terminology Criteria for Adverse Events (CTCAE version 5.0) based on their description in the diagnostic report.

### Statistical analysis

Descriptive data were compared with the t-test or the Fisher’s exact test, as appropriate, and continuous variables were reported using means and standard deviations (SD). Sensitivity, specificity and likelihood ratios (LR + = Sensitivity/(1-Specificity) and LR- = (1-Sensitivity)/Specificity) of the edrophonium test were calculated for the overall cohort as well as for MG subgroups, and according to the RNS test results and current treatment with pyridostigmine at the time of the edrophonium test (pyridostigmine was withheld on the day of the edrophonium test). Bonferroni correction was performed post-hoc for multiple comparisons of sensitivity values. A logistic regression model was utilised to assess the effects of sex, age at onset, disease duration, tested symptom, RNS test result, antibody status, and MG subtype by affected region on the result of the edrophonium test in patients with MG, and odds ratios (OR) with 95% confidence intervals (95% CI) were reported. Model robustness of the logistic regression model was assessed using the events-per-variable ratio (EPV), multicollinearity diagnostics with generalised variance inflation factors (GVIF), and influence diagnostics with Cook’s distance, as well as a sensitivity analysis by excluding potentially influential observations.

No imputation of missing data was performed. A p-value of < 0.05 was considered statistically significant. Statistical analysis was conducted using R version 4.4.1 (R Project for Statistical Computing), R Studio (version 2024.09.0 + 375, RStudio PBC) and Prism version 10.6.0 (GraphPad Software, Boston MA, USA).

## Results

### Study cohort characteristics

**Fig. 1:**
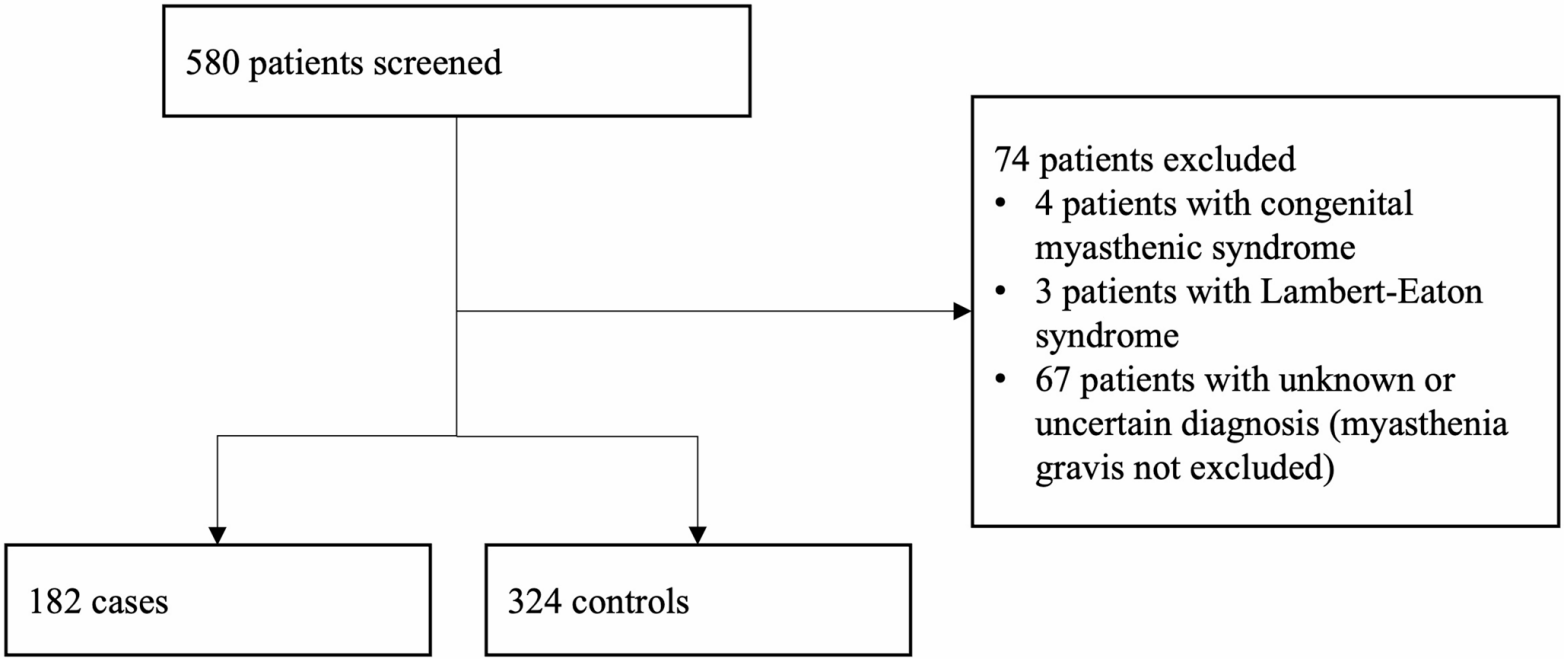
Inclusion flow chart

We screened 580 patients with available edrophonium test results (Fig. 1). 74 patients were excluded, 67 of whom had an uncertain or unknown diagnosis, 4 patients were diagnosed with a congenital myasthenic syndrome, and 3 patients with Lambert-Eaton syndrome. MG was diagnosed in 182 patients, who were compared to 324 controls (Table 1). Female patients accounted for 41.2% of the MG group and 55.2% of the control group, and the mean ages at the edrophonium test were 55.8 years (SD 19.3) and 53.6 years (SD 16.9), respectively. Early onset AChR-MG was diagnosed in 38 (20.9%), and late onset AChR-MG in 79 patients (43.4%). Age at onset was unknown in 16 patients (8.8%) with AChR-MG and thymoma-associated MG was diagnosed in 13 patients (7.1%). Six patients (3.3%) had antibodies against MuSK, while only one patient (0.5%) was positive for LRP4 antibodies. SNMG was reported in 29 patients (15.9%). All patients in the SNMG subgroup were tested for antibodies against AChR in RIA, which was confirmed with a CBA in 7 patients (24.1%). In 17 patients (58.6%) with SNMG testing for antibodies against MuSK was performed (in 5 patients ELISA and CBA, in 9 patients only ELISA, and in 3 patients only CBA), while CBA was additionally used to test for antibodies against LRP4 in 7 patients (24.1%). At the time of the edrophonium test, 109 patients (59.9%) had pure ocular (MGFA I) symptoms, and 73 patients (40.1%) had generalised (MGFA II-IV) muscle weakness. Pyridostigmine was prescribed to 29 patients (15.9%) at the time of the edrophonium test, while only 7 patients (3.8%) received immunosuppression. Among cardiac and pulmonary co-morbidities, arterial hypertension was the most common, reported in 43 patients (23.6%) in the MG group and 46 patients (14.2%) in the control group.

**Table 1:** Patient characteristics

	Cases, *n* = 182	Controls, *n* = 324	*p*-value
**Sex (%)**			
Female	75 (41.2)	179 (55.2)	0.0030
Male	107 (58.8)	145 (44.8)	
**Mean age at MG onset (SD)**	53.2 (20.1)	-	-
**Edrophonium test**			
Mean age at edrophonium test (SD)	55.8 (19.3)	53.6 (16.9)	0.1861
Tested symptom (%)			
Ocular	160 (87.9)	287 (88.6)	0.8854
Only non-ocular	22 (12.1)	37 (11.4)	
Negative results (%)	34 (18.7)	284 (87.7)	< 0.0001
Positive results (%)	148 (81.3)	40 (12.3)	
Minimal response	28 (18.9)	17 (42.5)	0.0021
Moderate/strong response	101 (68.3)	17 (42.5)	
Unknown	19 (12.8)	6 (15.0)	
**Adverse events (%)**			
No	153 (84.1)	295 (91.0)	0.0203
Yes	29 (15.9)	29 (9.0)	
CTCAE grade I	27/29 (93.1)	26/29 (89.7)	> 0.9999
CTCAE grade II	2/29 (6.9)	2/29 (6.9)	
CTCAE grade III	0/29 (0.0)	1/ (3.4)	
**Repetitive Nerve Stimulation (%)**			
Negative	54 (29.7)	226 (69.8)	< 0.0001
Positive	95 (52.2)	8 (2.4)	
Not performed	33 (18.1)	90 (27.8)	
**MG subgroup (%)**			
Early onset (< 50 years) AChR-MG	38 (20.9)	-	-
Late onset (≥ 50 years) AChR-MG	79 (43.4)	-	
Thymoma-associated AChR-MG	13 (7.1)	-	
AChR-MG with unknown age at onset	16 (8.8)	-	
MuSK-MG	6 (3.3)	-	
LRP4-MG	1 (0.5)	-	
SNMG	29 (15.9)	-	
**MG subtype by symptoms at the time of the edrophonium test (%)**			
Ocular	109 (59.9)	-	-
Generalised	73 (40.1)	-	
**MGFA classification (%)**			
MGFA I	109 (59.9)	-	-
MGFA II	53 (29.1)	-	
MGFA III	17 (9.3)	-	
MGFA IV	3 (1.7)	-	
**Treatment (%)**			
Pyridostigmine	29 (15.9)	-	-
Immunosuppression	7 (3.8)	-	
Unknown	20 (11.0)	-	
**Cardiac co-morbidities (%)**			
Arterial hypertension	43 (23.6)	46 (14.2)	0.1346
Coronary heart disease	1 (0.5)	8 (2.5)	
Atrial fibrillation	2 (1.1)	4 (1.2)	
Congestive heart failure	1 (0.5)	0 (0.0)	
Past myocardial infarction	1 (0.5)	4 (1.2)	
Hyperkinetic heart syndrome	0 (0.0)	1 (0.3)	
Dilated cardiomyopathy	0 (0.0)	1 (0.3)	
Rheumatoid myocarditis	0 (0.0)	1 (0.3)	
**Pulmonary co-morbidities (%)**			
Bronchial asthma	2 (1.1)	2 (0.6)	0.6772
Chronic obstructive pulmonary disease	2 (1.1)	4 (1.2)	
Obstructive sleep apnoea	3 (1.6)	0 (0.0)	
Chronic bronchitis	1 (0.5)	1 (0.3)	
Lung tumour	2 (1.1)	1 (0.3)	
Past tuberculosis with lung involvement	1 (0.5)	1 (0.3)	

AChR=Acetylcholine Receptor. CTCAE=Common Terminology Criteria for Adverse Events. LRP4 =Low-density Lipoprotein-related Protein 4. MG=Myasthenia Gravis. MGFA=Myasthenia Gravis Foundation of America. MuSK=Muscle Specific Kinase. SNMG=Seronegative Myasthenia Gravis

### Diagnostic performance of the edrophonium test

**Fig. 2:**
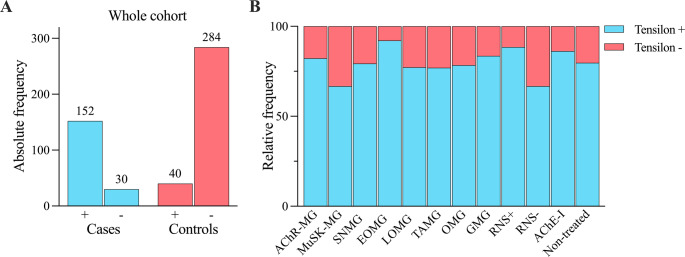
**A** Absolute frequencies of positive (+) and negative (-) edrophonium test results in the whole cohort. **B** Relative frequencies of edrophonium test results in various MG subgroups. (see Table 2 for sensitivity values). AChE-I=Acetylcholine esterase inhibitor (treatment with pyridostigmine). AChR=Acetylcholine Receptor. EOMG=Early-onset Myasthenia Gravis. GMG=Generalised Myasthenia Gravis. LOMG=Late-onset Myasthenia Gravis. MG=Myasthenia Gravis. MuSK=Muscle Specific Kinase. OMG=Ocular Myasthenia Gravis. TAMG=Thymoma-associated Myasthenia Gravis. RNS+= Positive Repetitive Nerve Stimulation. RNS-=Negative Repetitive Nerve Stimulation. SNMG=Seronegative Myasthenia Gravis

The sensitivity and specificity of the edrophonium test in the whole MG cohort were 83.5% and 87.7%, respectively, with an LR + of 6.77 and an LR- of 0.19 (Fig. 2A; Table 2). No significant differences were found between sensitivity values of AChR-MG, MuSK-MG, and SNMG, early onset AChR-MG and late onset AChR-MG, ocular MG and generalised MG, or untreated patients and patients under pyridostigmine treatment (Fig. 2B). Notably, in 51/73 patients (69.9%) with generalised MG, only ocular symptoms were assessed in the edrophonium test, among whom 44 (86.3%) had a clinical response. In 18/22 patients (81.8%) assessment of bulbar or limb weakness yielded a positive test result. The sensitivity of the edrophonium test was higher in patients with a decrement after RNS compared to patients without a decrement (88.4% [LR + 7.16, LR- 0.13] vs. 66.7% [LR + 5.40, LR- 0.38], respectively; *p* = 0.0110). In 12 of 34 patients with MG (35.3%) in whom the edrophonium test was negative, no decrement was observed on RNS. Among all patients with a positive edrophonium test result, a significantly larger proportion of patients with MG had a strong response to edrophonium compared to the control group (101/129 patients, 78.3% vs. 17/34 patients, 50.0%; *p* = 0.0021). Alternative diagnoses of patients in the control group with a positive edrophonium test result are listed in supplementary Table 2.

**Table 2:** Sensitivities and likelihood ratios of the edrophonium test in the whole cohort and predefined MG subgroups

	LR+	LR-	Sensitivity (%)	*p*-value of Fisher’s exact test (corrected)
Whole cohort	6.77	0.19	83.5	-
AChR-MG	6.66	0.20	82.2	> 0.9999
MuSK-MG	5.40	0.38	66.7	
SNMG	6.42	0.24	79.3	
Early onset AChR-MG	7.46	0.09	92.1	0.3520
Late onset AChR-MG	6.25	0.26	77.2	
Thymoma-associated MG	6.23	0.26	76.9	-
Ocular MG	6.35	0.25	78.4	> 0.9999
Generalised MG	6.77	0.19	83.6	
Positive RNS	7.16	0.13	88.4	0.0110
Negative RNS	5.40	0.38	66.7	
Pyridostigmine treatment	6.98	0.16	86.2	> 0.9999
No treatment with AChE-I	6.46	0.23	79.7	

AChE-I=Acetylcholine Esterase Inhibitor. AChR=Acetylcholine Receptor. LR + = Positive Likelihood Ratio. LR-=Negative Likelihood Ratio. MG=Myasthenia Gravis. MuSK=Muscle Specific Kinase. RNS=Repetitive Nerve Stimulation. SNMG=Seronegative Myasthenia Gravis

Frequencies of positive results (i.e. sensitivities) of the edrophonium test were compared between subgroups using the Fisher’s exact test. The distinction between ocular and generalised MG was based on symptoms present at the time of the edrophonium test

In the multivariable logistic regression model (EPV = 13), MG patients with a positive RNS result had significantly increased odds of a clinical response to edrophonium (OR 3.79, 95% CI 1.48–10.33, *p* = 0.0067). The presence of antibodies against MuSK, compared to AChR, was associated with lower odds of a positive edrophonium test (OR 0.08, 95% CI 0.01–0.82, *p* = 0.0254). Sex, age at onset, disease duration, tested symptom, and MG subtype by affected region did not affect the result of the edrophonium test significantly. Multicollinearity was low for all covariates (GVIF^1/(2df)^ < 1.25) and no observation showed extreme influence (maximum Cook’s distance = 0.088). However, sensitivity analysis revealed limited robustness of the effect of the antibody subtype.

### Safety of the edrophonium test

AEs were reported in 58 patients (11.5% of the whole cohort), most of whom (53/58 patients, 91.4%) had only mild events (CTCAE grade I; Table 1). Moderate AEs (CTCAE grade II) were observed in 4/58 patients (6.9%) including presyncope in one patient which was transient and self-limiting. A severe AE (bradycardia; CTCAE grade III) occurred in one patient (1/58, 1.7%) without known cardiac co-morbidities, in whom intervention with atropine led to a fast reversal. The most common AEs in the whole cohort were vertigo (16/506 patients, 3.2%), muscle cramping or fasciculations (13/506 patients, 2.6%), hyperhidrosis (12/506 patients, 2.4%), lacrimation (11/506 patients, 2.2%), and nausea (11/506 patients, 2.2%). No cases of respiratory insufficiency were observed in our cohort (Table 3).

**Table 3:** Adverse events. Relative frequencies refer to the whole cohort (*n* = 506)

	Number of patients (%)
Total number of patients with adverse events	58 (11.5)
Vertigo	16 (3.2)
Fasciculations or muscle cramping	13 (2.6)
Hyperhidrosis	12 (2.4)
Lacrimation	11 (2.2)
Nausea	11 (2.2)
Sialorrhea	8 (1.9)
Blurred or double vision	4 (0.8)
Hot flash	4 (0.8)
Emesis	3 (0.6)
Abdominal pain	2 (0.4)
Anxiety or restlessness	2 (0.4)
Bradycardia requiring atropine administration	1 (0.2)
Presyncope	1 (0.2)
Flush	1 (0.2)
Accommodation dysfunction	1 (0.2)

## Discussion

In this large case-control study, we report the diagnostic performance and safety of the edrophonium test in a large and well-defined patient cohort comprising multiple MG subtypes. Our main finding is that the edrophonium test can serve as a valuable adjunctive tool for diagnosing MG.

To date, few studies on the performance of the edrophonium test in diagnosing MG have been published. Nicholson et al. reported a sensitivity of 88% for generalised MG and 92% for ocular MG, respectively, with a specificity of 97% [18]. However, a positive edrophonium test result was used as an inclusion criterion in some patients, so overestimation due to incorporation bias cannot be ruled out [14]. By contrast, we observed a slightly lower sensitivity of 83.5% and specificity of 87.7% in our cohort. A comprehensive review calculated a pooled LR + of 15.0 and LR- of 0.11 for anticholinesterase tests [24], compared to an LR + of 6.77 and an LR- of 0.19 in our study. Many of the included studies, however, were limited by small sample size or their study design [18, 20, 21, 25–28]. Variations of the edrophonium test, such as the swallowing-edrophonium test or the Edrophonium-Hess test, have also been used successfully in patients with purely bulbar or ocular symptoms with high diagnostic accuracy [22, 29]. A recent prospective study demonstrated a higher predictive value of the edrophonium test for ocular MG (sensitivity 94%, specificity 90%, AUC 0.91) compared to antibody testing, RNS, SFEMG, or the ice pack test [19].

Antibody testing and RNS or SFEMG are considered the gold standard for the diagnosis of MG [10–13, 15, 30, 31]. Nevertheless, sensitivity varies by disease severity and MG subtype, with markedly lower values in ocular MG [13, 14, 16]. For example, we recently reported RNS sensitivity values of 71.6% for generalised MG but only 38.5% for ocular MG, respectively [16]. Sensitivity was considerably higher for the edrophonium test in our cohort, with values reaching approximately 80% in both groups. Additionally, while sensitivities were similar in the AChR-MG- (82.2%) and SNMG-subgroups (79.3), fewer MuSK-MG patients responded to edrophonium (66.7%), which is in line with the clinically observed poorer response to treatment with acetylcholinesterase inhibitors in this group [32]. Unsurprisingly, MG patients with a positive RNS were more likely to have a positive edrophonium test. Notably, 66.7% of patients with MG and a negative RNS result still had a positive edrophonium test. Combining RNS and the edrophonium test might therefore increase their diagnostic performance in patients with suspected MG while antibody results are pending.

The edrophonium test was safe in our study. Adverse events were reported in 11.5% of the total cohort, most of which were mild. However, one patient required atropine, highlighting the need to monitor patients during and after the test. Patients are routinely asked about potential contraindications such as known cardiac or pulmonary comorbidities before the test at our centre, which presumably led to the exclusion of most patients with risk factors. Importantly, no severe adverse events — such as respiratory insufficiency or cardiac arrests — were documented. In the literature, one study reported several severe adverse events (e.g., asystole, severe respiratory insufficiency, seizures or a transient ischemic attack) [33], but those results were based solely on questionnaires in which physicians retrospectively estimated the number of tests performed and the complication rates. Other studies have reported only mild muscarinic adverse events [22, 34, 35].

Our study has some limitations that should be acknowledged. Firstly, the retrospective study design of our study may have introduced information bias. Very mild, transient adverse events (e.g., mild hypersalivation), for example, may not have been documented, so the true number of adverse events is likely higher. Furthermore, not all patients in the SNMG subgroup were tested for antibodies against MuSK or LRP4, which likely reflects the restricted availability of antibody assays during a substantial proportion of the study period. Secondly, in most patients with generalised MG only ocular symptoms were tested, most likely reflecting a selection bias. Although, we were not able to stratify among this subgroup based on generalised symptoms, we found similar sensitivities in patients in whom ocular symptoms were evaluated and patients in whom only generalised symptoms were tested. Thirdly, interrater variability and observer bias may have influenced edrophonium test results, especially in cases in which minimal clinal response may have been classified as negative. This limitation becomes particularly pertinent given the absence of objective or standardised criteria for grading positive test responses and the inability to retrospectively assess interrater agreement of our data, as we relied on historical clinical reports from over 30 years. Despite this limitation, which applies to the MG as well as the control groups, our analysis in this large cohort indicate that a moderate to strong response is more likely associated with MG. Finally, our main logistic regression model was limited by the instability of the effect estimate for the antibody status, likely due to the small samples size in the MuSK-MG subgroup, and should therefore be interpreted cautiously.

A general limitation of the edrophonium test is the restricted availability of the drug in many countries, not only following the discontinuation of the test by the U.S. Food and Drug Administration (FDA) in 2018 due to concerns regarding safety and false-positive results [36], but also because of import shortages in many countries (e.g., in Europe). Nevertheless, we believe that the edrophonium test retains substantial clinical relevance. Where its use is not possible, the neostigmine test may serve as an alternative [37].

Current guidelines, such as those of the German Neurological Society, emphasise a multimodal diagnostic approach, combining clinical assessment, antibody testing, and neurophysiological studies, while acknowledging the diagnostic challenges in specific subgroups [2]. In our diagnostic workflow, the edrophonium test was performed early after clinical suspicion of MG, typically on the same day as RNS, and before the availability of antibody results. This sequence allows for rapid integration of neurophysiological and pharmacological information, which we consider particularly useful in ocular MG, where RNS has a lower sensitivity [16]. Additionally, our findings suggest that the edrophonium test may represent an informative tool for obtaining clinically relevant evidence in the diagnosis of SNMG. Moreover, in settings where RNS (or SFEMG) is not readily available, pharmacological testing may be especially feasible in acute cases under appropriate monitoring conditions.

## Conclusion

In conclusion, we provide robust real-world evidence for the diagnostic performance and safety of the edrophonium test in a large and heterogeneous cohort of patients with myasthenia gravis. Our results support its role as an adjunctive diagnostic tool, particularly in ocular MG, where the sensitivity of RNS and antibody testing is lower, as well as in cases where rapid information is needed to guide treatment decisions in severely affected patients for whom RNS is not available and antibody results are pending.

## Supplementary Information

Supplementary Material 1

## Data Availability

No patient data or study related documents are shared within this paper. Reasonable requests from qualified investigators will be considered by the corresponding author in accordance with applicable privacy regulations.

## Abbreviations

95% CI: 95% Confidence Interval
AChE-I: Acetylcholine Esterase Inhibitor
AChR: Acetylcholine Receptor
AE: Adverse Event
CBA: Cell-based Assay
CTCAE: Common Terminology Criteria for Adverse Events
ELISA: Enzyme-linked Immunosorbent Assay
EOMG: Early-onset Myasthenia Gravis
EPV: Events-per-Variable Ratio
FDA: Food and Drug Administration
GMG: Generalised Myasthenia Gravis
GVIF: Generalised Variance Inflation Factor
LOMG: Late-onset Myasthenia Gravis
LR+: Positive Likelihood Ratio
LR-: Negative Likelihood Ratio
LRP4: Low-density Lipoprotein-related Protein 4
mg: Milligram
MG: Myasthenia Gravis
MGFA: Myasthenia Gravis Foundation of America
MuSK: Muscle Specific Kinase
nmol/l: Nanomole per Litre
OMG: Ocular Myasthenia Gravis
OR: Odds Ratio
RNS: Repetitive Nerve Stimulation
SD: Standard Deviation
SFEMG: Single-Fibre Electromyography
SNMG: Seronegative Myasthenia Gravis
TMG: Thymoma-associated Myasthenia Gravis
