# Supplementary material for: Diagnostic performance and safety of the edrophonium test in myasthenia gravis: a retrospective case-control study
Source: Neurol Res Pract. 2026 Feb 9;8(1):11. doi: 10.1186/s42466-026-00468-6 (PMC12888114; doi:10.1186/s42466-026-00468-6)
Supplement: Supplementary file 1 — Supplementary Material 1 [file 42466_2026_468_MOESM1_ESM.docx]

**Supplementary table 1** Alternative definite or suspected diagnoses in the control cohort (n=324).

|  | **Number of patients (%)** |
| --- | --- |
| Isolated third, fourth or sixth cranial nerve palsy | 76 (23.5) |
| Idiopathic ptosis | 72 (22.2) |
| Vascular CNS disorder (i.e. cerebral infarction or haemorrhage, etc.) | 26 (8.0) |
| Motor neuron disease | 17 (5.2) |
| Endocrine orbitopathy | 15 (4.6) |
| Strabismus | 14 (4.3) |
| Blepharospasm | 11 (3.4) |
| Chronic progressive external ophthalmoplegia | 9 (2.8) |
| Multiple sclerosis or clinically isolated syndrome | 9 (2.8) |
| Neuropathy (incl. CIDP, Miller-Fisher syndrome, etc.) | 8 (2.5) |
| Primary myopathy or dystrophy | 8 (2.5) |
| Dysphagia, dysarthria or dyspnoea due to non-neurological disorder | 8 (2.5) |
| Mass lesion in the CNS (i.e. cancer) | 7 (2.2) |
| Horner syndrome | 4 (1.2) |
| (Para-)infectious | 3 (0.9) |
| Parkinson’s disease | 3 (0.9) |
| Sicca syndrome | 2 (0.6) |
| Neuromyotonia | 1 (0.3) |
| Myotonic syndrome | 1 (0.3) |
| Susac syndrome | 1 (0.3) |
| Progressive supranuclear gaze palsy | 1 (0.3) |
| Sarcoidosis | 1 (0.3) |
| Recurrent painful ophthalmoplegic neuropathy | 1 (0.3) |
| Voice tremor | 1 (0.3) |
| Multiple system atrophy | 1 (0.3) |
| Hydrocephalus | 1 (0.3) |
| Connective tissue disease | 1 (0.3) |
| Thyrotoxic paralysis | 1 (0.3) |
| Ptosis as a complication after cataract operation | 1 (0.3) |
| Other disorders or symptoms not usually included in the differential diagnosis of MG (i.e. physiological asymmetry of palpebral fissures, chronic fatigue syndrome, etc.) | 20 (6.2) |

CIDP=Chronic Inflammatory Demyelinating Polyradiculoneuropathy. CNS=Central Nervous System. MG=Myasthenia Gravis

**Supplementary table 2** Alternative diagnoses of patients in the control cohort with a positive response to edrophonium (n=40).

|  | **Number of patients (%)** | **Distribution of patients by response (moderate/strong : mild : unknown)** |
| --- | --- | --- |
| Isolated third, fourth or sixth cranial nerve palsy | 16 (40.0) | 7:5:4 |
| Motor neuron disease | 4 (10.0) | 1:3:0 |
| Vascular CNS disorder (i.e. cerebral infarction or haemorrhage, etc.) | 3 (7.5) | 2:1:0 |
| Chronic progressive external ophthalmoplegia | 2 (5.0) | 1:1:0 |
| Primary myopathy or dystrophy | 2 (5.0) | 0:2:0 |
| Mass lesion in the CNS (i.e. cancer) | 2 (5.0) | 0:2:0 |
| Idiopathic ptosis | 2 (5.0) | 1:1:0 |
| Endocrine orbitopathy | 1 (2.5) | 1:0:0 |
| Dysphagia, dysarthria or dyspnoea due to non-neurological disorder | 1 (2.5) | 0:0:1 |
| Blepharospasm | 1 (2.5) | 1:0:0 |
| Sicca syndrome | 1 (2.5) | 0:1:0 |
| Miller Fisher syndrome | 1 (2.5) | 0:1:0 |
| Multiple sclerosis | 1 (2.5) | 0:0:1 |
| Myotonic syndrome | 1 (2.5) | 1:0:0 |
| Susac syndrome | 1 (2.5) | 1:0:0 |
| Connective tissue disease | 1 (2.5) | 1:0:0 |

CNS=Central Nervous System. MG=Myasthenia Gravis
